# Supplementary material for: Evaluation of In Vitro Production Capabilities of Indole Derivatives by Lactic Acid Bacteria
Source: Microorganisms. 2025 Jan 13;13(1):150. doi: 10.3390/microorganisms13010150 (PMC11767884; doi:10.3390/microorganisms13010150)
Supplement: Supplementary file 1 [file microorganisms-13-00150-s001.zip › FS.pdf]

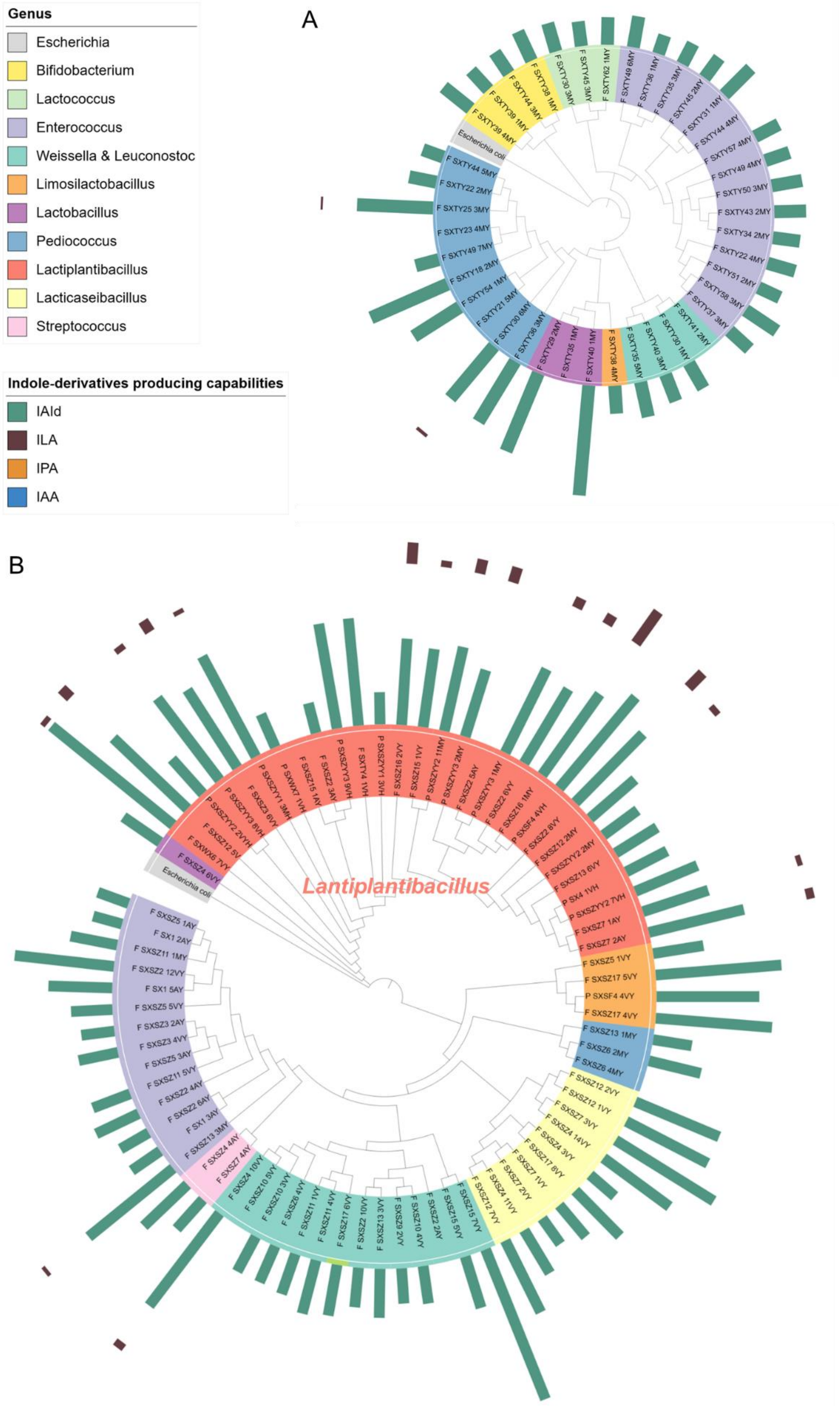

Figure S1. Phytological tree showing partial (A) and full-length (B) 16S rDNA gene sequences of LAB strains isolated from Shanxi Province and their indole derivative-producing capabilities.

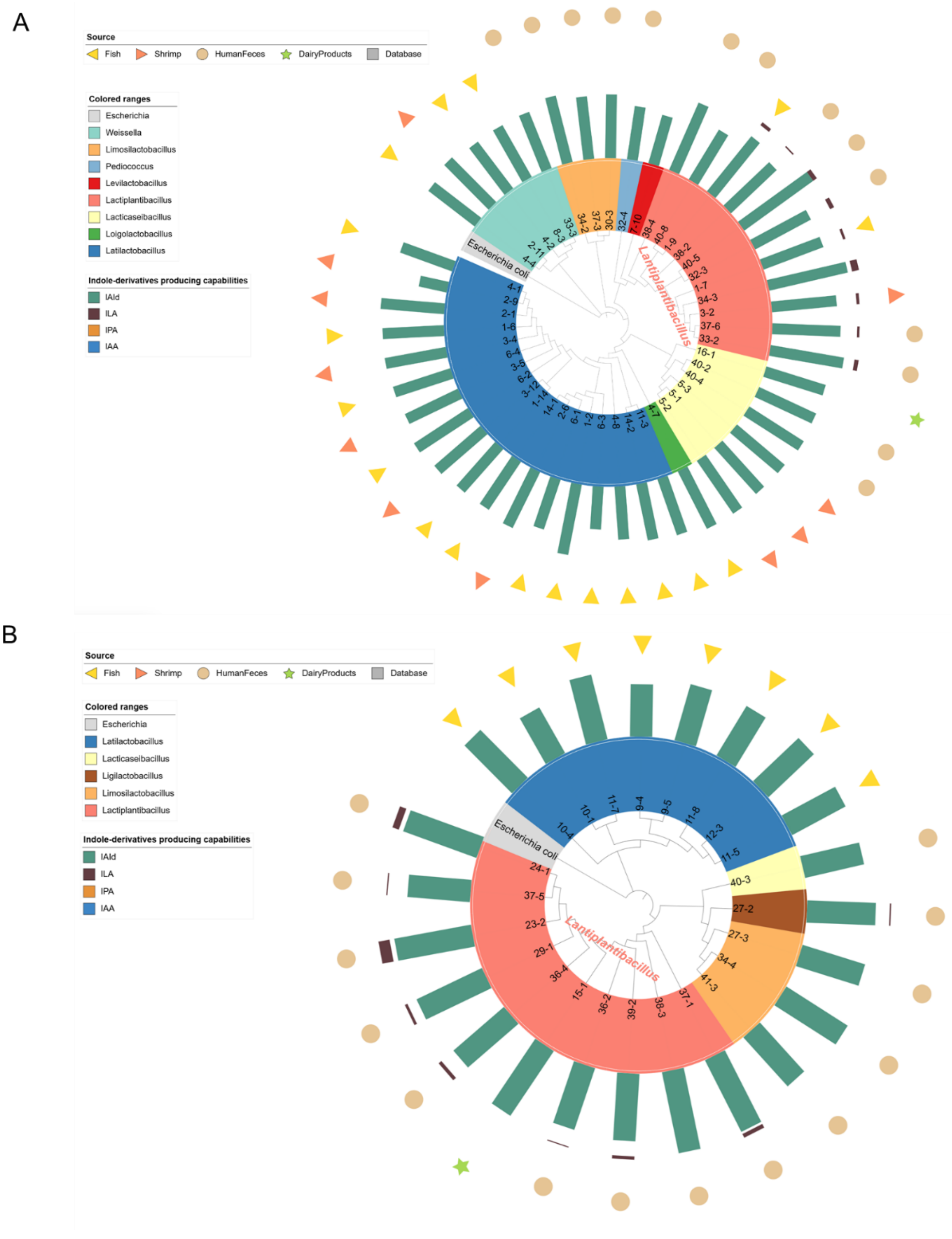

Figure S2: Phytological tree showing partial (A) and full-length (B) 16S rDNA gene sequences of LAB strains isolated from Jiangsu Province and their indole derivative-producing capabilities.
